# A Rare Case of Catastrophic Antiphospholipid Antibody Syndrome: A Case Report and Review of Traditional Cardiovascular Risk Factors Implicated in Disease Occurrence

**DOI:** 10.7759/cureus.7221

**Published:** 2020-03-09

**Authors:** Meghana Parsi, Maitreyee Rai, Ronald Swaab

**Affiliations:** 1 Internal Medicine, Crozer-Chester Medical Center, Upland, USA; 2 Internal Medicine, Crozer Chester Medical Center, Upland, USA; 3 Hematology - Oncology, Crozer Chester Medical Center, Upland, USA

**Keywords:** aps, caps, traditional cardiovascular risk factors, thrombosis

## Abstract

Antiphospholipid syndrome (APS) is a multisystem autoimmune condition characterized by recurrent thrombosis and/or recurrent pregnancy loss. Clinical manifestations include minor clots to clots involving multiple organ systems, termed catastrophic antiphospholipid syndrome (CAPS). The interaction of several autoantibodies, anti-beta-2-glycoprotein 1 antibodies, lupus anticoagulant, and anticardiolipin antibody with plasma proteins is associated with a heightened procoagulant state. As a result, physicians need to recognize this syndrome in a patient presenting with thrombosis in multiple organs. Not only this, but physicians must be aware of traditional cardiovascular risk factors that increase a patient's risk of atherosclerosis, such as diabetes, hypertension, hypercholesterolemia, and smoking that increase these patient's clot risk. Primary care doctors must be diligent in recognizing and aggressively controlling traditional risk factors to prevent further endothelial and vascular injury that can precipitate thrombosis. We present here a case of a 69-year-old female who presented with thrombosis in several organs, which proved to be secondary to CAPS. Unfortunately, she also had several cardiovascular risk factors that put her at an increased risk of clot formation and propagation. After the resolution of her acute thrombotic event, she was sent home on anticoagulation but returned with clot propagation.

## Introduction

Antiphospholipid syndrome (APS) is a rare autoimmune disease characterized by hypercoagulability and thrombosis in both the arterial and venous circulations, with or without pregnancy morbidity. This condition presents with various clinical manifestations, affecting multiple organs. Its most severe form, catastrophic antiphospholipid syndrome (CAPS), occurs in less than 1% of patients and is associated with thrombosis occurring in multiple organs. Deep venous thrombosis (DVT), pulmonary embolism (PE), and stroke are the most common manifestations [1]. The most commonly implicated antibodies include anti-beta-2-glycoprotein 1 antibodies, lupus anticoagulant, and anticardiolipin antibody. The main function of these antibodies is to serve primarily as anticoagulants. However, unlike their name implies, these antibodies are associated with a procoagulant state, rather than a bleeding diathesis. The binding of antiphospholipid (APL) to membrane plasma proteins upregulates a cascade of interactions among complement, platelets, endothelial cells, and adhesion molecules, leading to a prothrombotic state. While the prevailing idea maintains that thrombosis is the main pathogenic phenomenon in APS, the role of atherosclerosis must not be forgotten. Since the underlying thrombophilia cannot be prevented in APS, recognition and control of risk factors involved in atherosclerosis can greatly reduce the future risk and burden of disease in these individuals. We present here a case of CAPS with an extensive atherothrombotic disease in a 69-year-old Hispanic woman.

## Case presentation

A 69-year-old Hispanic female with a past medical history of hypertension, hyperlipidemia, diabetes mellitus type 2, and peripheral vascular disease requiring a right lower extremity below-knee amputation presented with gradual onset of right upper extremity arm pain and chest pain. A review of systems was positive for a productive cough, which had developed a week ago. On exam, the patient was febrile with a temperature of 100.6°F and the right upper extremity was exquisitely tender to palpation. There were normal heart sounds with decreased breath sounds and dullness to percussion on the cardiopulmonary exam. Her initial labs are included in Table 1.

**Table 1:** Significant labs of day of admission

Parameter (normal range)	Labs on day of admission
Hemoglobin (11.6-15)	9.2 g/dL
White blood cell (WBC) (4.8-10.8)	17,000 cells/mm^3 ^
Procalcitonin (<0.5)	5.72 mg/L
Lactic acid (0.5-1.9)	2.3 mmol/L
Troponin (<0.5)	1.47 ng/mL
Blood glucose	>400 mg/dL
Hemoglobin A1c (<5.6%)	10.1%

**Figure 1:**
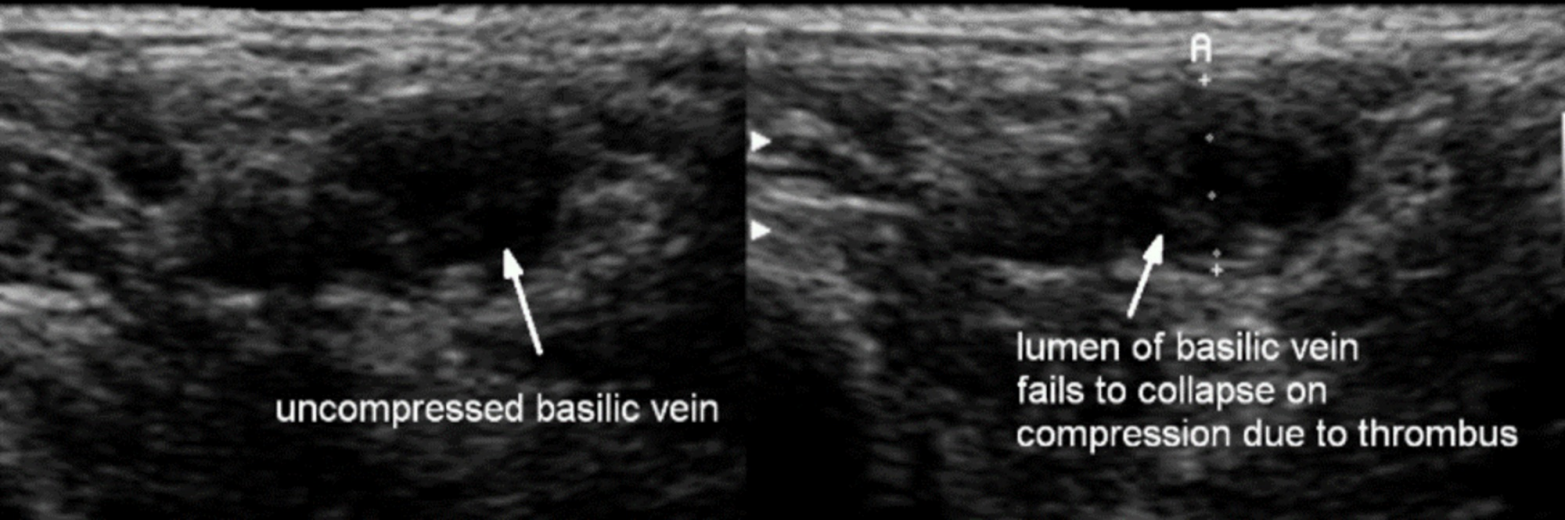
Venous Duplex Scan of the Upper Extremity The panel on the left shows a basilic vein that is not compressed by the ultrasound probe. In the right panel, the vein is compressed by the ultrasound probe; however, due to the presence of a clot within the lumen of the vein, the lumen is not collapsible (arrow).

Duplex imaging of the right upper extremity showed a partially occlusive DVT of the axillary and brachial veins with a superficial thrombus involving the right basilic and cephalic veins (Figure 1).

**Figure 2:**
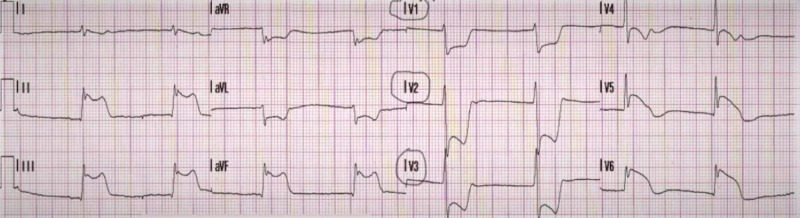
12-lead electrocardiogram (ECG) ST-segment depressions are seen in leads V1-V3 (circled). When the ECG leads are placed on the front of the chest, the presence of ST-segment depressions in these leads represents mirror images of the posterior wall of the heart. When depressions in leads V1-V3 are seen, one must be suspicious of the presence of a posterior wall myocardial infarction. This should prompt repeating another ECG, but with the leads placed in the back of the heart.

**Figure 3:**
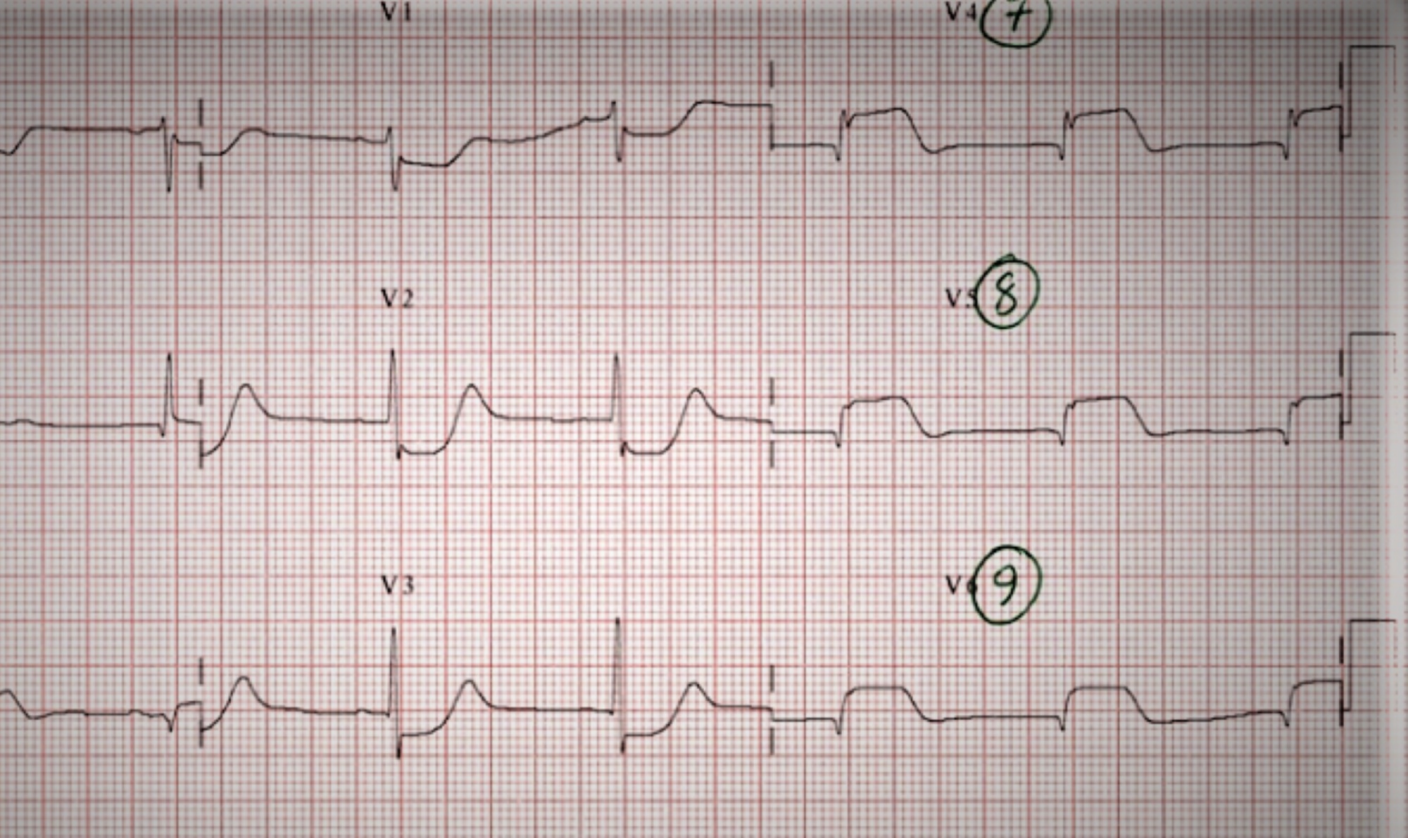
A posterior wall electrocardiogram (ECG) The presence of ST-segment depressions in leads V1-V3 prompted a posterior lead ECG. Instead of the leads being placed in the front of the chest, the leads were placed on the back of the chest. The above ECG shows ST-segment elevations in leads V7-V9 (circled), which confirms the diagnosis of a posterior wall myocardial infarction.

A CT scan of the chest revealed multiple patchy airspace opacities suspicious for multilobar pneumonia, without evidence of pulmonary embolus. An initial electrocardiogram (ECG) revealed ST-segment depressions in leads V1-V4, which was concerning for possible posterior wall myocardial infarction. A posterior lead ECG confirmed the diagnosis, with ST-segment elevations in leads V7-V9, confirming a posterior wall myocardial infarction (STEMI) (Figures 2, 3).

**Figure 4:**
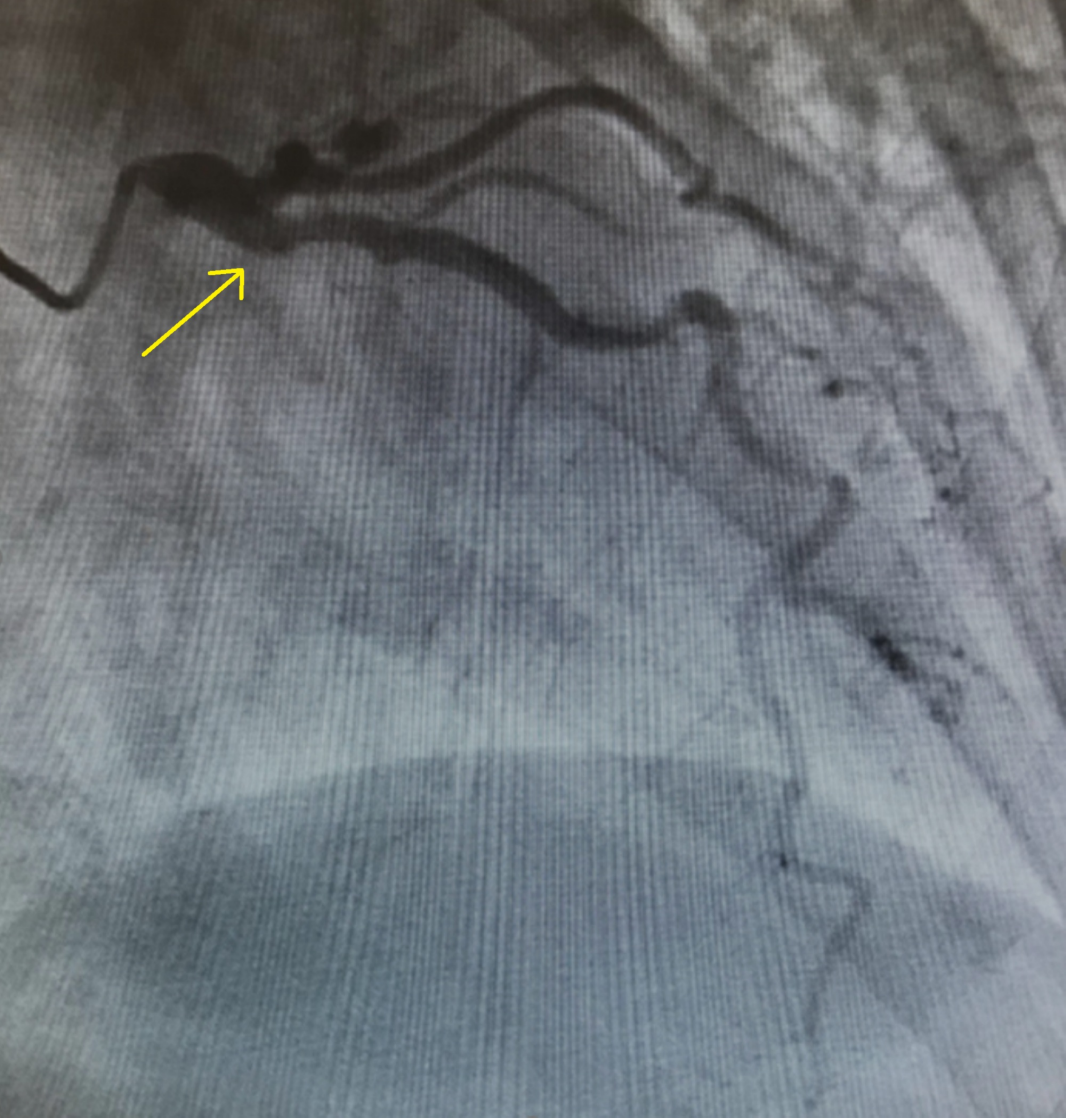
Left coronary angiogram Coronary angiogram showing a lack of flow in the region of the ostium of the left circumflex artery (LCX) (yellow arrow pointing to the origin of the LCX).

The patient was subsequently heparinized in the setting of STEMI and taken urgently to the cardiac cath lab, which revealed a tubular 100% thrombotic occlusion at the ostium of the left circumflex artery, for which a drug-eluting stent was placed (Figure 4).

The patient was started on intravenous antibiotics for pneumonia and started on appropriate dual-antiplatelet therapy and anticoagulation for myocardial infarction and DVT, respectively.

The patient’s hospital course was subsequently complicated by acute hypoxic respiratory failure requiring intubation for airway protection. An echocardiogram revealed new ischemic cardiomyopathy with an ejection fraction of 25%-30%, with akinesis of the entire inferolateral and inferior wall. One week into admission, the patient developed acute painless blackish discoloration of the left middle finger. The discoloration progressed to dry gangrene of the finger extending to the base of the middle phalanx. There was no associated cellulitis but required amputation. This was thought to be an embolic phenomenon in the setting of multiple thromboses due to an underlying hypercoagulable state. Immunological studies were positive for antinuclear antibody (1:160, speckled), elevated beta-2 glycoprotein IgG (>150; reference range <20 SAU), cardiolipin IgG antibody (>120; reference range <14 GPL), phosphatidylserine IgG (>100; reference range <10 U/ml), and lupus anticoagulant. In the setting of multiple thrombotic events, a presumptive diagnosis of CAPS was made. The patient was treated with steroids and five cycles of plasmapheresis. The patient was eventually bridged to warfarin before discharge. Repeat immunological studies revealed improved, but still revealed an elevated anticardiolipin, APL, and lupus anticoagulant.

The patient returned one month later after suffering from another thrombotic event, with acute occlusion of the right internal jugular vein in the setting of a subtherapeutic international normalized ratio (INR). She was sent home on an increased dose of warfarin with strict instructions for close follow-up with hematology/oncology.

## Discussion

APS is a rare autoimmune disease characterized by hypercoagulability and thrombosis in both the arterial and venous circulations, with or without pregnancy-related morbidity. This condition presents with various clinical manifestations, affecting multiple organs. Its most severe form, CAPS, occurs in less than 1% of patients and is associated with thrombosis occurring in multiple organs. The histopathological confirmation of the acute thrombotic microangiopathy in at least one organ or tissue forms one of the diagnostic criteria for CAPS. The diagnostic criteria for APS are presented in Table 2.

**Table 2:** Diagnostic criteria for CAPS APL, antiphospholipid; CAPS, catastrophic antiphospholipid syndrome

The diagnostic criteria for CAPS
Three or more organs, systems, and/or tissue involvement.
Development of manifestations in less than a week.
Histopathological evidence of intravascular thrombosis in at least one organ or tissue.
Presence of APL antibodies (lupus anticoagulant, anticardiolipin antibodies, and/or anti-beta-2-glycoprotein I antibodies) on two different occasions six weeks apart.

Definitive CAPS diagnosis
Presence of all four diagnostic criteria in a patient as listed above.
Probable CAPS diagnosis.
Presence of all four criteria, except for only two organs, systems, and/or tissue are involved .
Presence of all four criteria, except for the APL antibodies assay at least six weeks apart could not be done due to the early death of a patient who was never tested for APL before.
Criteria 1, 2, and 4 above.
Criteria 1, 3, and 4, with the third event occurring in more than a week but less than a month, despite anticoagulation.

Some of the earliest cases of CAPS date back to 1984; however, it was only in 1992 that Dr. Ronald Asherson defined it as an APL-related widespread coagulopathy. Hence, it is also known as Ronald Asherson syndrome [2]. The underlying mechanism in APS can involve an imbalance between the generation and lysis of fibrin, or the cytokine cascade as in systemic inflammatory response syndrome. As per the most recent CAPS registry, in the majority of cases (about two-thirds), there is an identifiable trigger that precipitates the acute thrombotic events. For example, infection, cancer and chemotherapy, surgery, subtherapeutic INR, and even pregnancy are all common causes [3].

APS is characterized by the development of thrombosis in the form of DVT, pulmonary thromboembolism, and stroke. The cardiac manifestations of APS include mainly valvular disease and intracardiac thrombus formation. Myocardial infarction occurs only 4% of the time [4]. Autoimmune disorders, including systemic lupus erythematosus, rheumatoid arthritis, systemic sclerosis, and APS, are known states of chronic inflammation that induce premature atherosclerosis. While the involvement of APL antibodies in the pathogenesis of thrombosis in APS is very much established in the literature, the presence of certain cardiovascular risk factors or medical conditions in these patients raises their risk for thrombosis. Recognizing and controlling these risk factors can help establish an effective treatment plan and prevent future thrombotic events.

These traditional cardiovascular risk factors for atherosclerosis include obesity, diabetes mellitus, hypertension, hyperlipidemia, and smoking. These factors are thought to act on inflammatory mechanisms and lipid metabolism within the body, leading to vascular injury and the propagation of atherosclerotic plaques. Rebeiro and Carvalho conducted a study in which they compared the presence of traditional risk factors in 39 patients with primary APS and those with secondary APS (an association with another immune disorder, such as lupus, infection or the use of medications such as chlorpromazine or procainamide). They revealed that in patients with primary APS, 46.2% had hypertension, 12.8% had diabetes mellitus, 28.2% had hypercholesterolemia and high levels of low-density lipoprotein (LDL), 15.4% had dyslipidemia, 84.6% had low levels of high-density lipoprotein, and 15.4% had a history of smoking [5]. In another study conducted by Kravvariti et al., the risk for atherosclerosis was compared between patients with primary and secondary APS and controls. The authors used ultrasonographic methods to compare the intima-media thickness in each of these patients. Overall, there was a higher incidence of plaques detected in the carotid and femoral arteries of primary and secondary APS patients (28% and 30%, respectively), compared to controls (9%) [6]. A study by Souza et al. demonstrated a strong association between hypertension and the presence of arterial thrombotic events in patients with APS. Compared to controls, those with primary APS had a disproportionately increased number of traditional risk factors [7]. Multiple studies have been conducted to delineate the association between diabetes and APS. One study demonstrated a higher level of APL antibodies in the sera of diabetic patients, suggesting an increased risk of microangiopathic complications. Diabetes is considered an atherogenic disease in which high levels of glucose activate NFκB, leading to monocyte adhesion, macrophage uptake of lipids, and the formation of fatty streaks [8]. The association between smoking and atherosclerosis is very much studied. Nicotine and carbon monoxide present in cigarette smoke results in vasoconstriction leading to vascular endothelial damage, setting the stage for the plaque build-up. An article published by Caldas et al. demonstrated an increased mortality rate in obese patients with APS. In this study, APS individuals with a higher body mas index (BMI) of >30 were found to have a greater incidence of life-threatening complications, such as PE, compared to APS individuals with a lower BMI of <30 [9]. Other reasons for premature atherosclerosis include the presence of atherosclerosis inducing APL antibodies, mainly lupus anticoagulant, anticardiolipin antibody, IgG antibodies against beta-2-glycoprotein antibody, and prothrombin. They are involved in the oxidation of LDL leading to endothelial dysfunction, monocyte adhesion, cytokine recruitment, intimal thickening, and subsequent narrowing of blood vessels.

## Conclusions

Overall, it has been proven time and time again that traditional cardiovascular risk factors greatly increase the thrombotic and atherosclerotic risk. Patients with APS already have a high thrombotic burden with the presence of APL antibodies and chronic inflammation leading to premature atherosclerosis. Primary care doctors must be diligent in recognizing and aggressively controlling traditional risk factors to prevent further endothelial and vascular injury. Strict lifestyle modifications in the form of diet and exercise are important to control diabetes, hypertension, and obesity. Moreover, silent myocardial infarction is a recognized entity in the elderly, women, and individuals with diabetes. Cessation of smoking is another modality that will halt the progression of atherosclerotic disease. A multidisciplinary approach involving primary care doctors, hematologists, and cardiologists is needed for integrated management of these patients to ensure improved patient outcomes.

## References

[1] Kazzaz NM, McCune WJ, Knight JS (2016). Treatment of catastrophic antiphospholipid syndrome. Curr Opin Rheumatol.

[2] Shoenfeld Y, Cervera R (2008). Asherson’s syndrome of the catastrophic antiphospholipid antibodies. J Rheumatol.

[3] Rand JH (2002). Molecular pathogenesis of antiphospholipid syndrome. Circ Res.

[4] George D, Erkan D (2009). Antiphospholipid syndrome. Prog Cardiovasc Dis.

[5] Ribeiro AR, Carvalho JF (2010). Traditional risk factors for cardiovascular disease in primary antiphospholipid syndrome (APS) when compared with secondary APS: a study with 96 patients. Acta Reumatol Port.

[6] Kravvariti E, Konstantonis G, Tentolouris N, Sfikakis PP, Tektonidou MG (2018). Carotid and femoral atherosclerosis in antiphospholipid syndrome: equivalent risk with diabetes mellitus in a case-control study. Semin Arthritis Rheum.

[7] Souza AWS, Silva NP, Carvalho JF (2007). Impact of hypertension and hyperhomocysteinemia on arterial thrombosis in primary antiphospholipid syndrome. Lupus.

[8] Piga R, Naito Y, Kokura S, Handa O, Yoshikawa T (2007). Short-term high glucose exposure induces monocyteendothelial cells adhesion and transmigration by increasing VCAM-1 and MCP-1 expression in human aortic endothelial cells. Atherosclerosis.

[9] Caldas CA, da Mota LMH, de Carvalho JF (2011). Obesity in primary antiphospholipid syndrome is associated with worse outcome. Joint Bone Spine.

